# Diurnal Regulation of In Vivo Localization and CO_2_-Fixing Activity of Carboxysomes in *Synechococcus elongatus* PCC 7942

**DOI:** 10.3390/life10090169

**Published:** 2020-08-29

**Authors:** Yaqi Sun, Fang Huang, Gregory F. Dykes, Lu-Ning Liu

**Affiliations:** Institute of Systems, Molecular and Integrative Biology, University of Liverpool, Liverpool L69 7ZB, UK; Yaqi.Sun@liverpool.ac.uk (Y.S.); fang.huang@liverpool.ac.uk (F.H.); Gregory.Dykes@liverpool.ac.uk (G.F.D.)

**Keywords:** carboxysome, carbon fixation, circadian clock, cyanobacteria, confocal microscopy, diurnal regulation, environmental acclimation, *Synechococcus elongatus*

## Abstract

Carboxysomes are the specific CO_2_-fixing microcompartments in all cyanobacteria. Although it is known that the organization and subcellular localization of carboxysomes are dependent on external light conditions and are highly relevant to their functions, how carboxysome organization and function are actively orchestrated in natural diurnal cycles has remained elusive. Here, we explore the dynamic regulation of carboxysome positioning and carbon fixation in the model cyanobacterium *Synechococcus elongatus* PCC 7942 in response to diurnal light-dark cycles, using live-cell confocal imaging and Rubisco assays. We found that carboxysomes are prone to locate close to the central line along the short axis of the cell and exhibit a greater preference of polar distribution in the dark phase, coupled with a reduction in carbon fixation. Moreover, we show that deleting the gene encoding the circadian clock protein KaiA could lead to an increase in carboxysome numbers per cell and reduced portions of pole-located carboxysomes. Our study provides insight into the diurnal regulation of carbon fixation in cyanobacteria and the general cellular strategies of cyanobacteria living in natural habitat for environmental acclimation.

## 1. Introduction

The extraordinary ability of cyanobacteria to survive in diverse ecosystems and adapt to extremes of environmental stress is ascribed to their metabolic robustness and tunability [1]. As cyanobacterial cells rely directly on light for photosynthesis, their abilities to respond to changes in the environmental light conditions are indispensable [2,3,4,5,6,7,8,9,10]. A typical example is the natural diurnal cycles that synchronize to the rotation of the Earth. It has been shown that the expression of many genes and metabolic activities in cyanobacteria are subject to the circadian rhythm that are regulated by an intrinsic circadian clock [11,12]. This regulation is of physiological importance to improve fitness and facilitate adaptation to diurnal light-dark cycles [13,14,15,16,17]. However, most of laboratory studies on cyanobacterial physiology are still performed under constant light, given the limitations of practical operations and considerations [18].

Carboxysomes are the essential CO_2_-fixing microcompartments present in all cyanobacteria [19,20,21,22,23]. The carboxysome is composed of a polyhedral protein shell that encapsulates the CO_2_-fixing enzymes ribulose-1,5-bisphosphate carboxylase/oxygenase (Rubisco) and carbonic anhydrases, as well as structural proteins and chaperones [24,25,26,27,28,29]. Impairment of carboxysome formation led to complete loss of CO_2_ fixation ability in cyanobacterial cells grown in ambient air conditions [30]. The cyanobacterial CO_2_-concentrating mechanisms (CCM) also comprise bicarbonate transporters in the cytoplasmic membrane and thylakoid-integrated CO_2_-converting complexes that function in accumulation of bicarbonate in the cytoplasm and preventing CO_2_ leakage from the cell [31,32]. Elevated bicarbonate then diffuses passively into the carboxysome through the shell and is dehydrated by carbonic anhydrases to CO_2_ near Rubisco enzymes [33]. Overall, this CCM system concentrates CO_2_ around Rubisco up to 1000-fold, facilitating Rubisco carboxylation and inhibiting oxygenation that leads to “wasteful” photorespiration [23,34].

Spatial distribution of carboxysomes within the cyanobacterial cell is pivotal for carboxysome biogenesis, functionality, and inheritance. It has been shown that multiple carboxysomes are equally distributed along the longitudinal axis of the rod-shaped cells of cyanobacterium *Synechococcus elongatus* PCC 7942 (Syn7942) [35]. Recent work has further revealed the spatial dynamics of carboxysomes in *Synechococcus* sp. PCC 7002 and the role of cell poles in in carboxysome activity modification [36]. This equidistant carboxysome partitioning was initially found to be determined by cytoskeleton protein ParA [35] (also termed McdA [37]) and was recently revealed to be mediated by McdB that can interact with McdA and carboxysome shells [37]. The McdAB system is widespread among β-cyanobacteria [38]. Our recent studies revealed that the biosynthesis, organization, and regulation of carboxysomes in Syn7942 cells are highly sensitive to changes in light [28,39]. Increase in light intensity could accelerate carboxysome biosynthesis, resulting in a higher abundance of carboxysomes and enhanced carbon-fixation activities of Syn7942 cells [39]. It could also alter the protein stoichiometry, diameter, and mobility of carboxysomes in cells [28]. Moreover, we showed that the spatial organization of carboxysomes in Syn7942 is correlated with the redox state of photosynthetic electron transport chain [39], a key controller for circadian rhythm in light-dark cycles [40]. Based on these results and the findings revealing that the expression of carboxysome genes and their encoded proteins is diurnally oscillated [41,42,43], we question whether in vivo organization and function of carboxysomes in Syn7942 are regulated under diurnal light-dark cycles.

Here, we use live-cell confocal fluorescence imaging to probe the localization of fluorescently labeled carboxysomes in the model cyanobacterium Syn7942 that grow in diurnal cycles. Additionally, we assess the impact of the circadian clock on the carboxysome biosynthesis and distribution in Syn7942 without *kaiA* that encoded the essential protein to sustain oscillation of the circadian clock [17]. We also determine the real-time carbon fixation activities and capacities of Syn7942 cells under diurnal conditions using in vivo Rubisco assays. Our results shed light on the physiological regulation of carboxysome organization and functionality in cyanobacteria that grow in natural environment with regular light-dark cycles.

## 2. Materials and Methods

### 2.1. Strains, Generation of Constructs, and Culture Conditions

*Escherichia coli* (*E. coli*) DH5α and BW25113 strains were used to generate plasmids for homologous recombination in cyanobacteria through λ-red recombination system [44], as described in previous work [3,28,32,39]. In brief, the plasmid containing the target gene coding sequence, *eyfp* coding sequence and apramycin resistant operon as well as 1500 bp upstream/downstream sequences of the target gene amplified from WT Syn7942 genome, was transformed into *E. coli* to replace the endogenous gene via homologous recombination for fluorescence labeling. The plasmid containing the spectinomycin resistant operon and 1500 bp upstream/downstream sequences of the target gene was transformed into *E. coli* to replace the endogenous gene for gene deletion. The diagram of recombination is shown in Figure S1. The generated plasmids were extracted from *E. coli* and were then transformed into Syn7942. The successful modification and segregation status of Syn7942 strains were confirmed by polymerase chain reaction (PCR) with primers designed up/downstream of the modified region. Agarose gel electrophoresis was performed with the standardized amounts of PCR products. The RbcL-eYFP Syn7942 mutant was generated in previous studies [28], with the C-terminus of the Rubisco large subunit RbcL fused with enhanced yellow fluorescent protein (eYFP) after a 11 amino acid linker (LPGPELPGPGP), at the native chromosomal locus and under the control of endogenous promoter. All strains used were listed in Table S1. Primers used for construct generation and screening were listed in Table S2.

Syn7942 cells were cultivated in BG-11 medium [45] or on BG-11 agar plates with TES buffer pH 8.2 (10 mM C_6_H_15_NO_6_S) and sodium thiosulphate (20 mM Na_2_S_2_O_3_), solidified by 1.5% Agar-agar (*w*/*v*). For constant moderate light treatment (CL), 30 mL of cultures were added in filter capped culture flasks (Nunc^TM^ Cell Culture Treated EasYFlasks^TM^, Thermo-Fisher Scientific, Waltham, MA, USA) and kept in 30 °C culture room with constant shaking at 120 rpm under warm white growth light (3200 K) at an intensity of 50 μmole photons·m^−2^·s^−1^. Diurnal light treatments (DL) with 12-h dark and 12-h illumination were performed according to method previously described [46]. Light intensity and other parameters were kept the same with CL treatment. Antibiotics were supplied at following concentrations: apramycin at 50 μg·mL^−1^, spectinomycin at 50 μg·mL^−1^, and kanamycin at 50 μg·mL^−1^ and chloramphenicol at 10 μg·mL^−1^ in ethanol.

### 2.2. Carbon-Fixation Assays

The maximum CO_2_ fixation capacity measurement was carried out as described previously [28,39]. The in vivo carbon fixation rate measurements were carried out with BG-11 growth medium (nitrogen saturated via bubbling) containing 2 mM of radioactive sodium bicarbonate (NaH^14^CO_3_). Gas exchanges with the atmosphere were minimized in air-tight containers during assays. The cultures in sealed flasks were placed into light treatments for 30 min of growth. Cultures were sampled at a volume of 500 μL and mixed with 200 μL 10% formic acid. The mixture was then placed on heat blocks at 95 °C to remove unfixed NaH^14^CO_3_. The pellets were resuspended in distilled water then mixed with scintillation cocktail (Ultima Gold XR; PerkinElmer, Waltham, MA, USA). Radioactivity measurements were carried out using a scintillation counter (Tri-Carb; Perkin-Elmer, Waltham, MA, USA). Raw readings were processed to determine the amount of fixed ^14^C, calibrated by pre-permeabilized cell samples treated with mixed alkyltrimethylammonium bromide (MTA), and then converted as total carbon fixation rates. Carbon fixation rates were normalized by cell density, indicated by measured OD_750_ readings.

### 2.3. Circadian Bioluminescence Monitoring

Detection of bioluminescence from the luciferase reporters in Syn7942 was performed using a protocol adapted from previous work [47]. pAM2195 introduces the bioluminescence-generating genes *luxAB* and *luxCDE* with circadian-controlled *psbAI* promoter into the Neutral Insertion site II (NSII) of Syn7942 genome as described [47]. Successful pAM2195 transformant was inoculated in BG-11 medium for 2 days of initial growth with DL treatments. Cultures were then pipetted on solid medium (BG-11 1.5% agar) to form a droplet and then placed back to CL treatment for further growth before imaging. For signal quantification, the petri dish containing cell droplets was placed in a light-tight imaging box for bioluminescence capturing for 1 min manually by ImageQuant LAS 4000 (GE Healthcare Life Sciences, Waltham, MA, USA) with a 2-h imaging interval and placed back to CL before next imaging over a tracking period of 22 h.

### 2.4. Fluorescence Microscopy and Data Analysis

Sample preparation was done as described earlier [3]. For quantitative imaging, laser scanning confocal microscopy used a Zeiss LSM780 with alpha Plan-Fluor 100 × 1.45 Oil objective and excitation at 514 nm from Argon laser. Emissions of YFP signal were captured at 520–550 nm. Chlorophyll auto-fluorescence signals were captured at 660–700 nm. Images were recorded as 512 × 512 pixels images in 16 bits. KaiA-eYFP/RbcL-CFP dual fluorescence imaging was performed as described in [27]. The sample platform was pre-incubated and thermo-controlled at 30 °C before and during imaging. The laser power and imaging settings were maintained the same for all samples for quantitative comparison of fluorescence signals. Images were captured with all pixels below saturation.

Intensity profiling, carboxysome recognition was carried out using Fiji (ImageJ 1.52p, National Institute of Mental Health, Bethesda, MD, USA) [48]. Raw data were processed by Origin 2018 (OriginLab, Northampton, MA, USA) and MATLAB R2018a (Mathworks, Natick, MA, USA) for profile extraction and statistical analysis and the goodness-of-fit parameter for Violin plot visualization. Image SXM [49] was used for statistical analysis of carboxysome numbers per cell, carboxysome distribution within cells, as well as dimensions of cell length/width measurements, as performed previously [39]. Carboxysome distribution profiles along the longitudinal axis and short axis of the cells were analyzed following the method described previously [39]. Analysis of standard deviation of the distribution profiles along the longitudinal axis was performed according to [2,3]. To evaluate the effectiveness of sampling, sampling errors were calculated from three randomized sub-dataset at each timepoint. For each timepoint, a minimum of 300 cells was analyzed. Differences were analyzed with two-sided student *t*-test for significance in pairs or one-way ANOVA and Tukey test for multiple-group comparison. Polar distribution frequency was analyzed based on [36] using ImageSXM. Ten per cent of cell length at both cell ends was considered as the polar region. Polar distribution frequencies were calculated as the percentage of carboxysomes that located in the polar region of all the cells analyzed at each timepoint during diurnal cycles.

## 3. Results

### 3.1. Carboxysome Biosynthesis Is Regulated during Diurnal Cycles in Syn7942

To determine whether carboxysome abundance and subcellular organization are regulated during diurnal cycles, we first made a Syn7942 mutant by transforming a luciferase reporter plasmid pAM2195 [47] into wild-type (WT) Syn7942 cells. The intensity profiles of luciferase bioluminescence exhibit a peak at 10–14 h during the 22 h period (Figure S2), consistent with previous findings [47]. This confirmed the proper DL treatments and the circadian regulation in Syn7942 under our established growth conditions. The cell dimensions are relatively constant within experimental error during DL (Figure S3).

**Figure 1:**
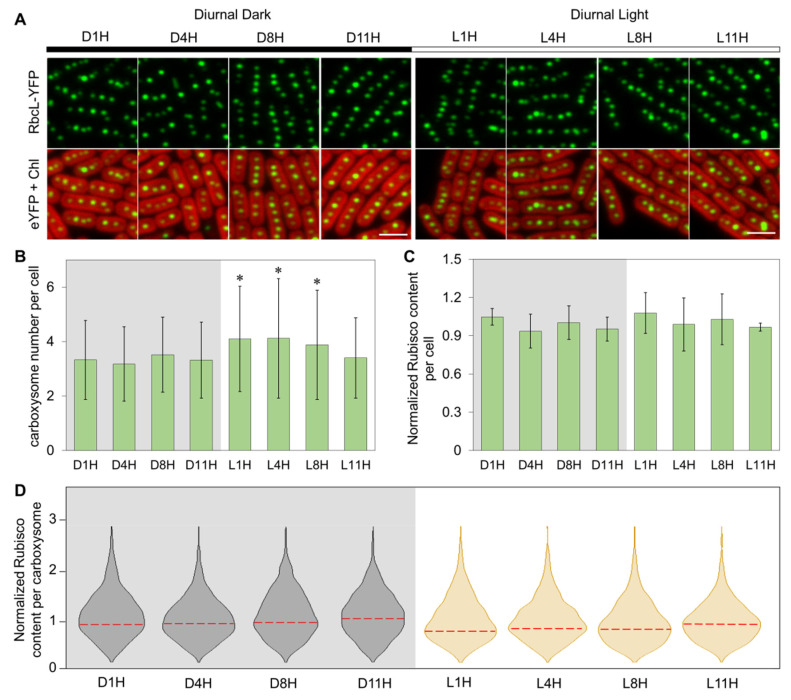
Carboxysome abundance and Rubisco content in Syn7942 cells grown during diurnal light-dark cycles. (**A**) Representative confocal images taken at respective time points during DL. Merged images show carboxysomes in green and Chl fluorescence in red. Scale bar = 2 μm. (**B**–**D**) Analysis of carboxysome number per cell, total Rubisco content per cell (estimated by RbcL-eYFP content from fluorescence microscopy), and Rubisco content per carboxysome during the dark-light cycle in A. Violin plots were generated by R illustrate the fluorescence intensity distribution of RbcL-eYFP during selected time points. The representative values and deviations were represented by Peak value from kernel density fitting and half-width at half maximum (HWHM). Error bars represent standard deviations. A total of 200 cells were analyzed for each timepoint. * *p* < 0.05.

We then grew the RbcL-eYFP Syn7942 cells under DL (Figure 1A). The *eyfp* gene was fused to the 3′-end of *rbcL* at the native chromosomal locus and under control of the endogenous promoter (Figure S1). This ensures that the proteins are expressed in context and at physiological levels [27,39]. We performed live-cell confocal imaging on the RbcL-eYFP Syn7942 strain at selected timepoints that covered 1 h before/after light transition as well as quarter marks in a cycle at 1H, 4H, 8H, and 11H from −24 h to 0 h, and then counted the carboxysome number per cell [39] (Figure 1B). A higher carboxysome number per cell was detected in the Syn7942 cells during the light period of diurnal cycles (4.1 ± 1.9 for L1H, 4.1 ± 2.2 for L4H, 3.9 ± 2.0 for L8H, *n* = 200 as cell counts for each timepoint) than those in the dark period (3.3 ± 1.5 for D1H, 3.2 ± 1.4 for D4H, 3.5 ± 1.4 for D8H, 3.3 ± 1.4 for D11H, *n* = 200 as cell counts for each timepoint) (Figure 1B, *p* < 0.05), except for L11H (3.4 ± 1.5 carboxysomes per cell, *n* = 200).

We also determined the contents of Rubisco in the RbcL-eYFP mutant during DL cycles, by quantifying the YFP signal per cell [27]. The cellular levels of Rubisco remain relatively constant (Figure 1C, *p* = 0.86, *n* = 200 as cell counts for each timepoint). The average Rubisco content per carboxysome in cells, as indicated by the peak value ± half-width at half-maximum (HWHM) [28], was relatively lower at L1H, L4H, and L8H than that determined at D1H, D4H, D8H, D11H, and L11H (0.77 ± 0.47, 0.83 ± 0.42, and 0.81 ± 0.51 compared with 0.94 ± 0.56, 0.94 ± 0.51, 0.96 ± 0.62, 1.05 ± 0.59, and 0.93 ± 0.47) (Figure 1D, *n* = 200 as cell counts for each timepoint).

### 3.2. Subcellular Localization of Carboxysomes Is Diurnally Regulated in Syn7942

**Figure 2:**
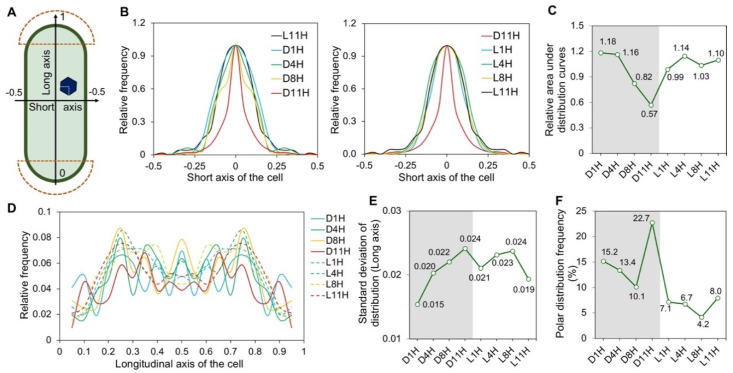
Carboxysome localization under diurnal light-dark conditions. (**A**) Diagram of the carboxysome localization analysis within the rod-shaped Syn7942 cells. Cell pole regions were marked in dash line, covering the 10% of cell length along the longitudinal axis from each end of the cell. (**B**) Distribution profiles of carboxysomes along the short axis of the cell. (**C**) Quantitative analysis of the area under the distribution profile curves in (**B**). (**D**) Distribution profiles of carboxysomes along the longitudinal axis of the cell. (**E**) Standard deviation (SD) analysis of the distribution profiles along the longitudinal axis in (**D**). (**F**) Polar distribution frequency of carboxysomes (located within the polar region marked in (**A**). For each timepoint, 200 cells were analyzed.

We evaluated the spatial localization of carboxysome within the DL-adapted cells (Figure 2A). Carboxysome distribution profiles along the short axis of the cell [28] and analysis of the relative areas under distribution curves indicated that at the later stages of the dark period (D8H and D11H), carboxysomes exhibit a more central positioning along the short axis of the cell, in contrast to the carboxysome distribution at other timepoints of DL cycles (Figure 2B,C).

Analysis of the distribution profiles of carboxysomes along the longitudinal axis of the cell (Figure 2D) and standard deviations of the distribution profiles showed that segregation of carboxysomes was reinforced during the dark period from D1H to D11H (Figure 2E). It appears that more random distribution occurred during the light-dark transition; after light adaptation, the carboxysomes are prone to be segregated to specific cellular positions along the longitudinal axis of the cell (Figure 2E). In addition, carboxysomes exhibit a greater preference of polar distribution during the dark period than during the light period (Figure 2F). At D11H, carboxysomes have the highest tendency to be positioned at the cell poles (polar distribution frequency = 22.7 ± 2.2%, as average ± SE), which have been suggested to be the biogenic sites of carboxysomes and which accommodate inactive carboxysomes [36,50,51].

### 3.3. KaiA Deletion Alters the Carboxysome Localization and Abundance in Syn7942

**Figure 3:**
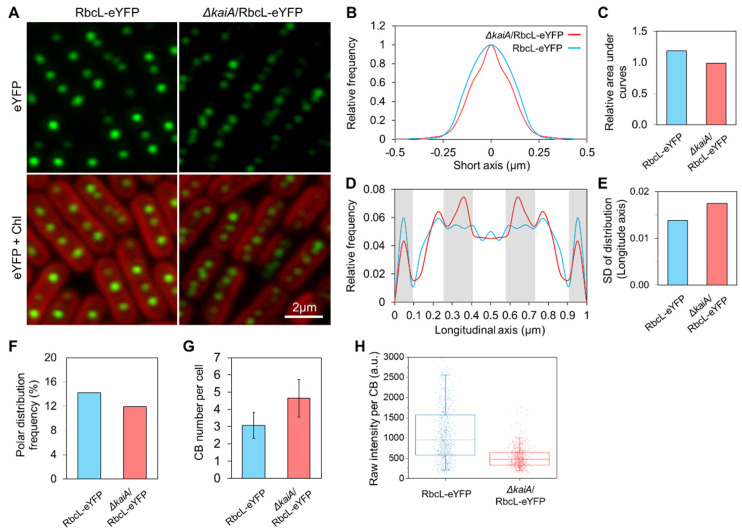
Carboxysome localization and abundance in the *∆kaiA* Syn7942 mutant. (**A**) Representative confocal images for Syn7942 RbcL-eYFP and *∆kaiA*/RbcL-eYFP cells grown in CL. Scale bar = 2 μm. (**B**,**C**) Carboxysome distribution profiles along the short axis of the cell and quantitative comparisons of the relative area under distribution profile curves. (**D**,**E**) Carboxysome distribution profiles along the longitudinal axis of the cell and quantitative comparisons of SD of distribution profiles. (**F**) Polar distribution frequency of carboxysomes. (**G**) Carboxysome (CB) number per cell measured from confocal images in A. Data are shown as mean ± SD. A total of 500 cells were analyzed for each strain in (**B**–**G**). (**H**) YFP Signal quantifications for on each carboxysome in two strains. *n* = 1000 as carboxysome number, *p* < 0.05. Data are shown in an arbitrary unit (a.u.). The averaging standard errors for Figure 3C,E,F are 0.13, 0.002, and 2.2, respectively.

To evaluate the regulation of circadian clock on carboxysome biogenesis in Syn7942 cells, we generated the circadian null strains, *∆kaiA* and *∆kaiA*/RbcL-eYFP, by deleting the core oscillator gene *kaiA* [52]. Successful deletion of *kaiA* was confirmed by PCR (Figure S4). We first characterized the carboxysome localization in the *∆kaiA* mutant in CL conditions (Figure 3). Confocal images were taken using the Syn7942 cells that have been fully adapted to CL for two days. It showed that both *∆kaiA*/RbcL-eYFP and RbcL-eYFP strains possess canonical carboxysome distributions (Figure 3A). However, the in-depth analysis revealed that carboxysomes in the *∆kaiA*/RbcL-eYFP mutant exhibited a relatively more centralized distribution along the short axis of the cell compared to the RbcL-eYFP strain (Figure 3B,C, *n* = 500 as cell counts for each strain). Carboxysomes possess more defined localization at specific regions along the longitudinal axis of the *∆kaiA*/RbcL-eYFP cell, compared with those in the RbcL-eYFP cell (Figure 3D,E, *n* = 500 as cell counts for each strain). Moreover, carboxysomes in the *∆kaiA*/RbcL-eYFP strain exhibited a lower tendency of the polar localization than in the RbcL-eYFP strain (Figure 3F).

Confocal image analysis also revealed that deletion of *kaiA* induced an increase in the copy number of carboxysomes (Figure 3G, 4.6 ± 1.1 in *∆kaiA*/RbcL-eYFP, 3.1 ± 0.8 in RbcL-eYFP, *n* = 500 as cell counts, *p* < 0.05). A ~1-fold decrease in the YFP fluorescence intensity per carboxysome was observed in *∆kaiA*/RbcL-eYFP (*n* = 1000 as carboxysome counts for each strain, *p* < 0.05) (Figure 3H), indicative of the reduced Rubisco content per carboxysome in the *∆kaiA*/RbcL-eYFP cell.

### 3.4. Carbon Fixation of Syn7942 Cells Is Rhythmically Alternated during Diurnal Cycles

**Figure 4:**
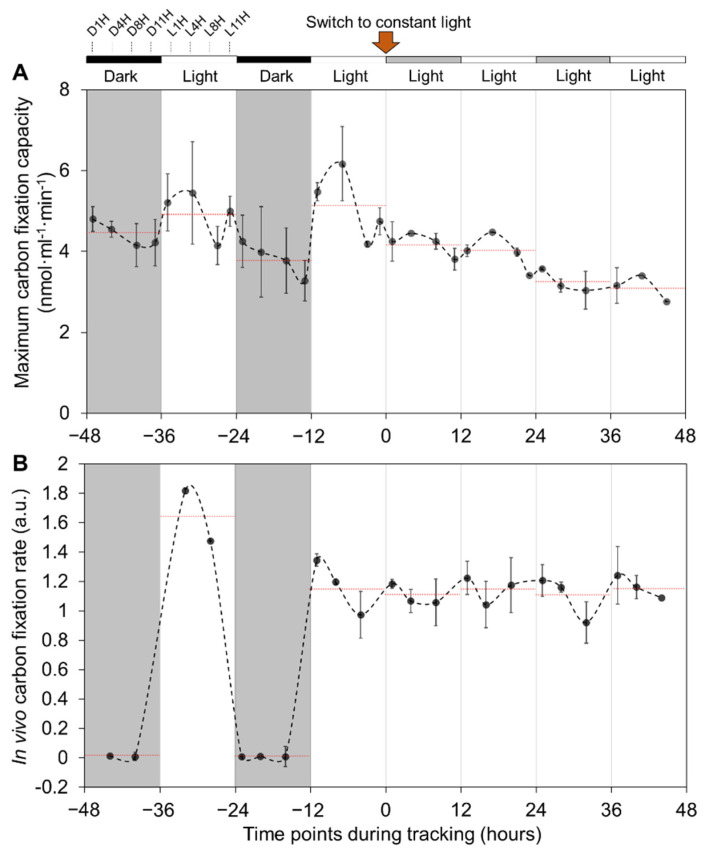
Maximum CO_2_-fixation capacities and in vivo CO_2_-fixation rates in WT Syn7942 under DL/CL conditions. (**A**) CO_2_-fixation capacities of cells grown in DL from −48 to 0 h and additional two days from 0 h to 48 h in CL. The black-white bars above and grey-white background indicate the dark and light cycles with corresponding time-point marks, respectively. Red arrow indicates the time-point of the switch from DL to CL. (**B**) In vivo ^14^C CO_2_-fixation rates measured during growth in NaH^14^CO_3_ containing BG-11 medium under DL and CL. Relative CO_2_-fixation rates are displayed in an arbitrary unit (a.u.). The red dashed lines indicate time point averages within the 12-h phase for fixation capacities and rates, respectively. Cell contents are normalized by cell density inferred through OD_750_ readings. Data are shown as mean ± SD. *n* = 3 (three independent biological replicates). Cell density OD_750_ was used for normalization.

To study the regulation of carbon fixation of Syn7942 cells under DL cycles, we measured the whole-cell maximum CO_2_-fixation capacities for four days (two days in DL and CL, respectively) using radioactive CO_2_-fixation assays (Figure 4A). Rhythmic changes in the CO_2_-fixation capacities were observed in DL: The cellular CO_2_-fixation capacities were gradually reduced during the dark periods from D1H to D11H and were then rescued suddenly after entering the light period (L1H) and sequentially reached the highest at L4H; the rise in CO_2_-fixation capacities was then followed by a decrease from the 2nd half of light period at L8H and L11H. Similar changes were also recorded in the 2nd DL cycle. On average, the whole-cell CO_2_-fixation capacities during the light period (5.0 ± 0.5 nmol·mL^−1^·min^−1^, *n* = 15) were higher (*p* < 0.05) than those during the dark period (4.1 ± 0.6 nmol·mL^−1^·min^−1^, *n* = 15). We further recorded the CO_2_-fixation capacities of Syn7942 cells when cells were transferred to CL (Figure 4A). The periodic variations of the cellular carbon fixation were retained during CL, including the increase at the initial timepoints of the subjective light periods during 12−24 and 37−40 h followed by a gradual daily decrease in CO_2_-fixation activities (daily averages as 4.65 ± 0.50 to 4.45 ± 0.94, 4.18 ± 0.36, and 3.18 ± 0.28, respectively). However, the average CO_2_-fixation activity during each subjective light period at CL was not elevated compared to that measured during the previous subjective dark period. These results indicate that both the circadian clock and light-dark transition play roles in the carbon-fixation regulation of Syn7942 cells.

**Figure 5:**
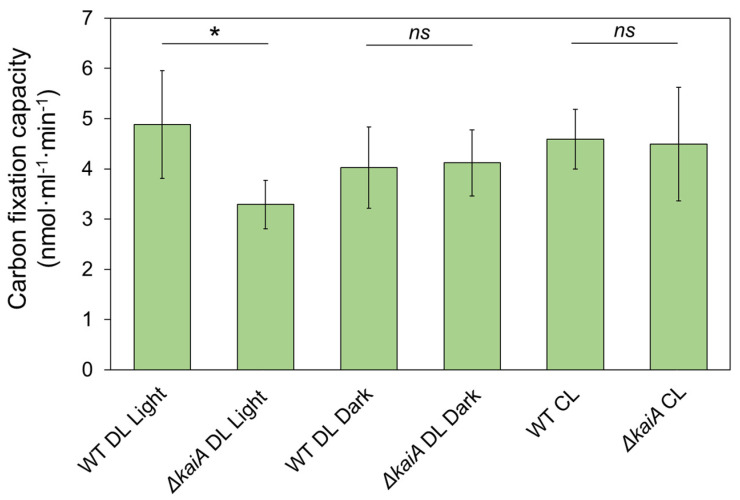
CO_2_-fixation assays of WT and *∆kaiA* Syn7942 cells during DL and CL conditions. Reduced CO_2_ fixation was detected in *∆kaiA* cells compared to WT during the light period of DL (*p* < 0.05). Error bar represents SD from a minimum of 4 independent biological replicates. Statistics for pair-wise comparison by Tukey test were shown in Table S3. (* as significant difference, *p* < 0.05; *ns* as not significant, *p* ≥ 0.05)

We also performed real-time in vivo CO_2_ fixation assays of Syn7942 cells in both DL and CL conditions (Figure 4B). Unlike the maximum CO_2_-fixation assays that were performed by adding exogenous ribulose 1,5-bisphosphate (RuBP) and bicarbonate at saturated concentrations to the permeabilized cells, in vivo CO_2_ fixation assays were conducted with endogenous RuBP and bicarbonate in Syn7942 cells. A notable decrease in the CO_2_-fixation rate (at a magnitude of ~100-fold) to almost zero was measured during diurnal dark periods (Figure 4B). After switching to CL, the average CO_2_-fixation rates of the cells became relatively constant regardless of the subjective light and dark periods (*p* = 0.19). Given that the maximum CO_2_-fixation assays indicated the functionality of these carboxysomes in Syn7942 cells (Figure 4A), in vivo CO_2_-fixation assays revealed that Syn7942 cells in the dark have a largely restricted Rubisco activity (Figure 4B), probably due to the limited levels of intracellular RuBP and bicarbonate in the dark-adapted Syn7942 cells. To address whether circadian clock is involved in the CO_2_-fixation regulation of Syn7942 during DL, we compared the Rubisco activities of *∆kaiA* and WT cells (Figure 5). The Rubisco activities of *∆kaiA* cells were significantly decreased during the light period of DL, in contrast to WT (*p* < 0.05, *n* = 4). No significant difference was detected between *∆kaiA* and WT during the dark period of DL and under CL (Table S3). The results implicated that circadian regulation on the CO_2_-fixation activities of carboxysomes specifically occurs during the light phase of DL.

## 4. Discussion

In this work, we characterized the effects of diurnal light-dark cycles on carboxysome biosynthesis, subcellular localization and function in Syn7942. We showed that Syn7942 cells adapt to diurnal cycles by orchestrating carboxysome abundance and spatial localization and CO_2_-fixation activities. Moreover, we evaluated the role of the circadian clock in regulating carboxysome biosynthesis and positioning using a *∆kaiA* Syn7942 mutant. Our results provide insight into the natural regulatory strategies evolved in cyanobacteria to control the assembly and functionality of carboxysomes, a key “biofactory” in global carbon fixation. A deeper understanding of the diurnal regulation of cyanobacterial metabolisms may also inform industrial applications to grow cyanobacteria that are facing the natural light-dark cycles in the outdoors [53].

Previous studies indicated that there were no significant changes in the percentage of tagged/non-tagged RbcL in the RbcL-eYFP strains grown under different light conditions [39]. The cellular levels of Rubisco detected in this study remain relatively constant under DL conditions (Figure 1C), in agreement with published proteomic data [54,55]. In contrast, transcriptional assays showed that the levels of cyanobacterial Rubisco genes *rbcL* and *rbcS*, together with other genes located in a *ccm* operon (*ccmK2*, *ccmL*, *ccmM*, *ccmN,* and *ccmO*), were rhythmically alternated under DL conditions [42,56]. This discrepancy may suggest possible post-transcriptional regulation of Rubisco [57]. Due to the imaging limit, it remains unclear whether free Rubisco proteins in the cytoplasm, as reported in marine cyanobacteria [58], were omitted in the cellular Rubisco quantification (Figure 1B,D), which merits future investigation.

Studies on the spatial localization of carboxysomes within the rod-shaped Syn7942 cells have suggested its significance in the biogenesis, function, and inheritance of carboxysomes [35,36,37,38]. Carboxysomes possess equal distribution along the longitudinal axis of the Syn7942 cell, which was indicated to be mediated by the McdAB system that is widespread among β-cyanobacteria [37,38]. Beyond these findings, here we showed the tunable subcellular positioning of carboxysomes during diurnal cycles, confirming the importance of light in determining the in vivo localization of carboxysomes. Under diurnal light-dark conditions, cell elongation and division of Syn7942 took place during the mid-phase of light period [59]. The activity of McdAB system might be determined by the cellular levels of ATP, which was known to accumulate throughout the light period [60]. Indeed, we observed gradually strengthened localization control from L1H to L8H, quantified as standard deviation of the distribution along the longitudinal axis of the cell (Figure 2E), which appear synchronously with the cell elongation and division events and rising levels of ATP. Carboxysomes and chromosomes are mutually exclusive in the cytoplasm [61]. Chromosome compaction mainly happened during the light period in the diurnal cycle, whereas during the dark period chromosomes were evenly distributed in the cytoplasm [62]. The restrained localization of carboxysomes in dark was therefore unlikely to be a result of space exclusion from chromosome positioning. The mechanisms that define the dynamic carboxysome distribution during diurnal cycles remain to be answered. In addition, we showed that carboxysomes have a high preference to locate at the cell poles during the dark phase in the RbcL-eYFP mutant; in the dark period, the WT Syn7942 cells show a reduced CO_2_-fixation activity (Figure 2F and Figure 4). These observations are consistent with the previous studies suggesting that the cell poles may serve as the sites for accommodating carboxysome precursors [50] or inactive or degrading carboxysomes [36].

In contrast to the better-understood distribution along the longitudinal axis of Syn7942 cells, the positioning of carboxysome along the short axis of the cell has remained poorly characterized. Our previous study has indicated that the central localization of carboxysomes along the short axis of the Syn7942 cell was ascribed to the reduced plastoquinone pool in photosynthetic electron transport chain [39]. The redox status is also a key signal in circadian regulation of cyanobacteria and can be modulated in light-dark transitions [63]. The plastoquinone pool is prone to be oxidized in the light phase [64] and cumulatively reduced throughout the major dark phase [65]. In agreement with this, we observed that a more central distribution of carboxysomes along the short axis of the cell appears in the dark period (Figure 2B,C), confirming the role of the redox state of the plastoquinone pool in mediating carboxysome positioning in Syn7942. Whether the circadian clock was involved in the redox-coupled positioning modulation of carboxysomes remains to be explored.

Interestingly, confocal images of the dual-labeled Syn7942 mutant KaiA-eYFP/RbcL-CFP (Figure S5) displayed that in addition to the polar localization of KaiA in Syn7942 as reported earlier [46], several KaiA fluorescent puncta are spatially close to carboxysomes in the cytoplasm (Figure S5A). Time-lapse imaging showed the dynamic formation process of KaiA assemblies in the dark period of DL (Figure S5B). The functional relevance of KaiA foci close to carboxysome in Syn7942 merits further investigations.

Our data suggested the regulation of circadian clock in carboxysome biogenesis and function in Syn7942 (Figure 3 and Figure 5). The circadian control of carbon assimilation has also been reported in higher plants [66,67,68]. In the chloroplast of dinoflagellates, Rubisco carboxylation is regulated by circadian clock through rearrangement of Rubiscos localization inside chloroplasts while maintaining constant levels of Rubisco proteins [57]. In C3 plants, the enzymatic activity of Rubisco is regulated by a series of Rubisco activases, of which the oscillated expression is circadian controlled [69,70]. In Crassulacean acid metabolism (CAM) plants, the CAM genes possess daily regulation by the circadian clock [71] and phosphoenolpyruvate carboxylase kinase represents the well-defined circadian control in primary CO_2_ fixation [72]. Meanwhile, the alternated distribution patterns of Rubisco in Syn7942 cells (higher numbers of carboxysomes that each contained fewer Rubisco), represented by RbcL-eYFP in *∆kaiA*/RbcL-eYFP, together with unchanged cellular levels of Rubiscos (data not shown) and carbon-fixation capacity compared to WT Syn7942 in CL (Figure 3 and Figure 5) might be an outcome of carboxysome compensation without KaiA to achieve similar levels of cellular carbon-fixation capacities, likely indicating the KaiA-involved modulation of carboxysomes [28]. It would be interesting in future research to survey the protein content of other carboxysome components to gain a complete picture of circadian clock-based structural plasticity. In conclusion, these studies highlighted the general regulation of carbon assimilation in cells in response to the natural diurnal cycles.

## Supplementary Materials

The following are available online at https://www.mdpi.com/2075-1729/10/9/169/s1, Figure S1: The strategy of fluorescent proteins (FPs) fusion and knock-out (KO) using REDIRECT protocol, Figure S2: Bioluminescence assays confirm the diurnal treatment and circadian control in the Syn7942 strain containing pAM2195, Figure S3: Cell dimensions of Syn7942 during DL, Figure S4: PCR screening of the segregation of *kaiA* mutants, Figure S5: Fluorescence images of the KaiA-eYFP/RbcL-CFP mutant show the distribution of carboxysomes and KaiA assemblies in Syn7942, Table S1: Strains used in this work, Table S2: Primers used in this work, Table S3: *p*-values of Tukey test on differences of maximum carbon fixation capacities listed in Figure 5.
