# Supplementary material for: Diurnal Regulation of In Vivo Localization and CO2-Fixing Activity of Carboxysomes in Synechococcus elongatus PCC 7942
Source: Life (Basel). 2020 Aug 29;10(9):169. doi: 10.3390/life10090169 (PMC7555275; doi:10.3390/life10090169)
Supplement: Supplementary file 1 [file life-10-00169-s001.pdf]

Supplementary Information

Diurnal Regulation of In Vivo Localization and CO_2-Fixing Activity of Carboxysomes in *Synechococcus Elongatus* PCC 7942

Yaqi Sun [1], Fang Huang [1], Gregory F. Dykes [1] and Lu-Ning Liu [1,*]

[1] Institute of Systems, Molecular and Integrative Biology, University of Liverpool, Liverpool L69 7ZB, United Kingdom

* Correspondence: luning.liu@liverpool.ac.uk (L.-N.L.)

Table S1. Strains used in this work.

Strains	Resistance	Description	Sources
RbcL-eYFP	Apramycin	Rubisco large subunit labelled with eYFP at the C-terminus	[1]
Δ*kaiA*	Spectinomycin	KaiA coding sequence replaced by spectinomycin resistant cassette	This work
Δ*kaiA*/RbcL-eYFP	Apramycin & Spectinomycin	Double mutant that was generated via two steps of transformation	This work
KaiA-eYFP	Apramycin	KaiA labelled with eYFP at the C-terminus	This work
RbcL-CFP	Kanamycin	Rubisco large subunit labelled with CFP at the C-terminus, served as competent cell for double mutant transformation	[2]
KaiA-eYFP/RbcL-CFP	Apramycin & Kanamycin	Double mutant that was generated via two steps of transformation	This work
pAM2195	Chloramphenicol	*luxAB* & *luxCDE* with circadian-controlled *psbAI* promoter inserted at Neutral Insertion site II (NSII)	[3]

Table S2. Primers used in this work.

Primers	Gene Sequences (5'-3')	Description
KaiA_F	GATCGCAGACAAAGTGAAGG	Amplify KaiA coding and 1500bp flanking sequences
KaiA_R	AAGAGGGTGAAGTCAGGTAG	
KaiA_FP_F	CTTTGTGAGATGTATCGACGGTCTATCCCACGAGAAACCCTGCCGGGCCCGGAGCTGCC	Amplify YFP & apramycin resistant cassette with 39 bp homolog sequence (underlined) adjunct to KaiA stop codon for YFP labelling
KaiA_FP_R	GAGAGAAATTGAGCCGAGCTTAAGACCTCCTTTACCTTTATTCCGGGGATCCGTCGACC	
KaiA_KO_F	TCTGTCTGCAGACTCAGTCCTGACAGGAGCGACTGCGTGATTCCGGGGATCCGTCGACC	Amplify spec cassette and 39 bp homolog sequence (underlined) to KaiA flanking sequence for Knock-Out
KaiA_KO_R	AGAAATTGAGCCGAGCTTAAGACCTCCTTTACCTTTTCATGTAGGCTGGAGCTGCTTC	
FKaiA KO SEG	ATGAGCTGCAGTGCTAGG	Screening segregation status of KaiA mutant in cyanobacteria
FKaiA FP SEG	CCGATGTTCCAGTCACCA	
RKaiA FP/KO SEG	TTACGAGGGCTCATACGC	

Table S3. *P*-values of Tukey test on differences of maximum carbon fixation capacities listed in Figure 5.

pairwise *p*-value	WT DL light	*ΔkaiA* DL Light	WT DL Dark	*ΔkaiA* DL Dark	WT CL	*ΔkaiA* CL
WT DL light	-	0.034*	0.117	0.660	0.992	0.971
ΔkaiA DL Light	0.034*	-	0.692	0.779	0.332	0.419
WT DL Dark	0.117	0.692	-	1.000	0.869	0.938
ΔkaiA DL Dark	0.660	0.779	1.000	-	0.975	0.991
WT CL	0.992	0.332	0.869	0.975	-	1.000
ΔkaiA CL	0.971	0.419	0.938	0.991	1.000	-

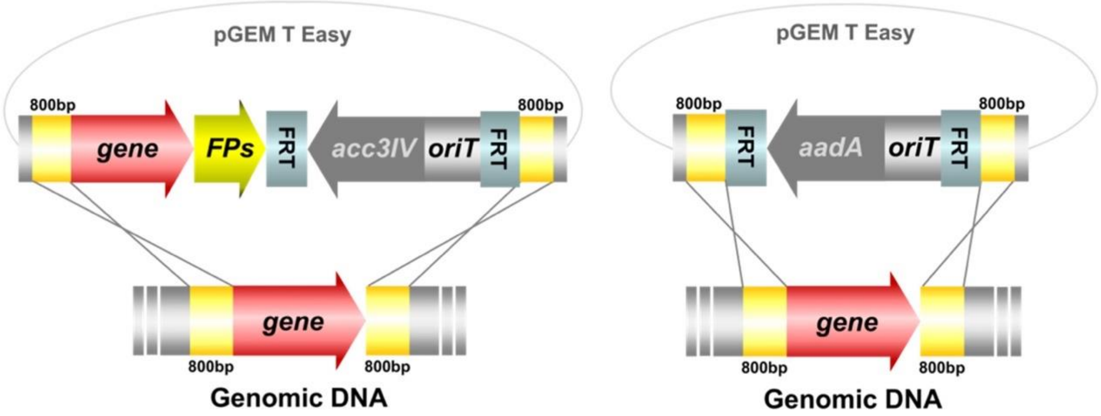

Figure S1. The strategy of fluorescent proteins (FPs) fusion and knock-out (KO) using REDIRECT protocol. A fragment contains 800 bp upstream and downstream of the gene with or without gene plus fluorescent protein together with an antibiotic-resistant cassette (encoded in reverse orientation) in order to replace the genomic DNA fragment by homologous recombination. FRT indicates flippase recognition target which can be used to excise inserted cassette. *acc3IV* and *aadA* indicate apramycin and spectinomycin resistant genes respectively for YFP labelling and KO mutants. OriT indicates short sequence as the origin of transfer during bacterial conjugation.

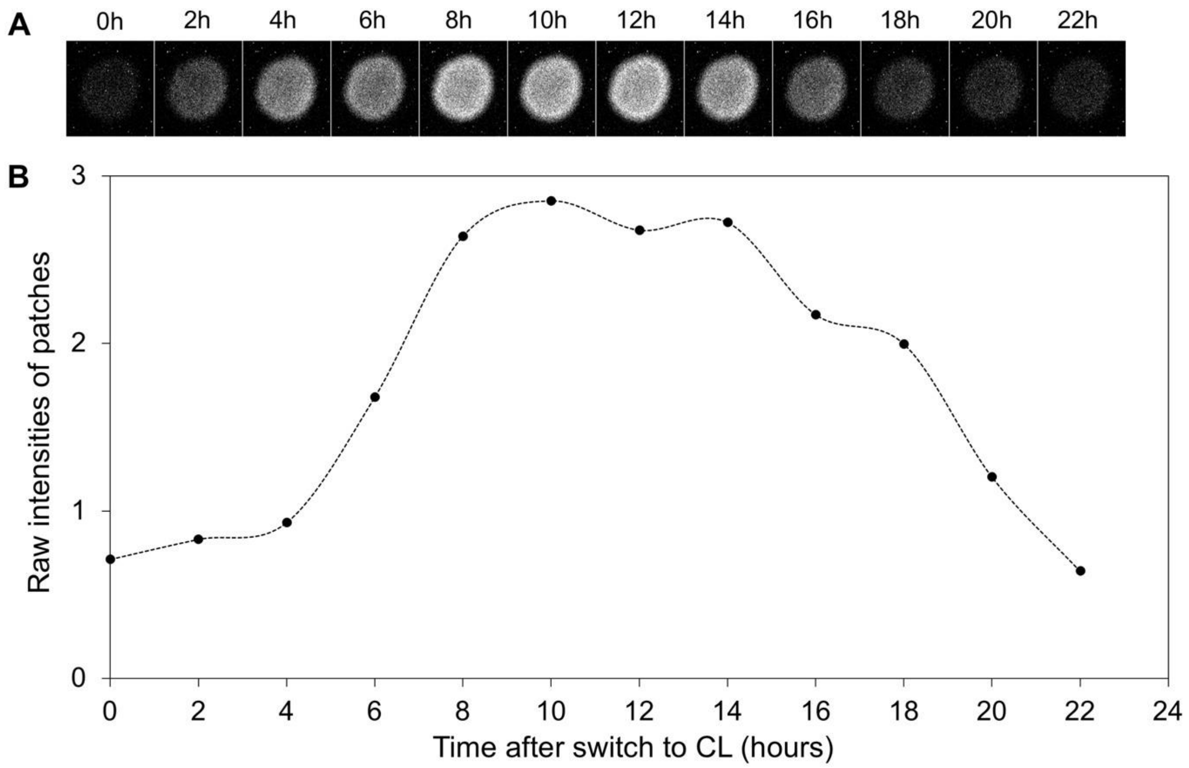

Figure S2. Bioluminescence assays confirm the diurnal treatment and circadian control in the Syn7942 strain containing pAM2195. A. Time-lapse image montage of pAM2195 Syn7942 cells containing the luciferase reporter vector pAM2195, grown under CL switched from the dark period of DL with two-hour intervals. B. Quantification of bioluminescence intensities of the cells during the time course.

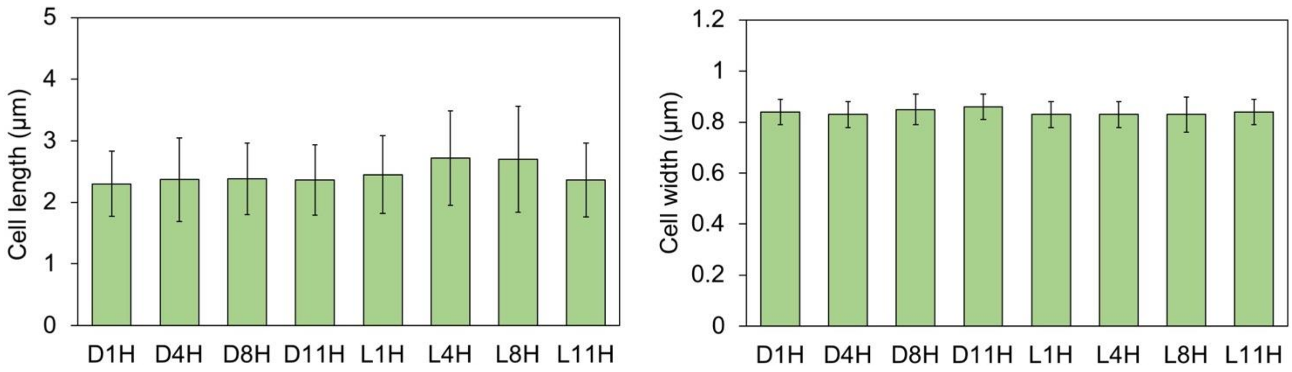

Figure S3. Cell dimensions of Syn7942 during DL. Average cell lengths (left) were similar within experimental error. Average cell widths (right) were comparable (n = 200 as cell counts for each timepoint). Error bars represent SD.

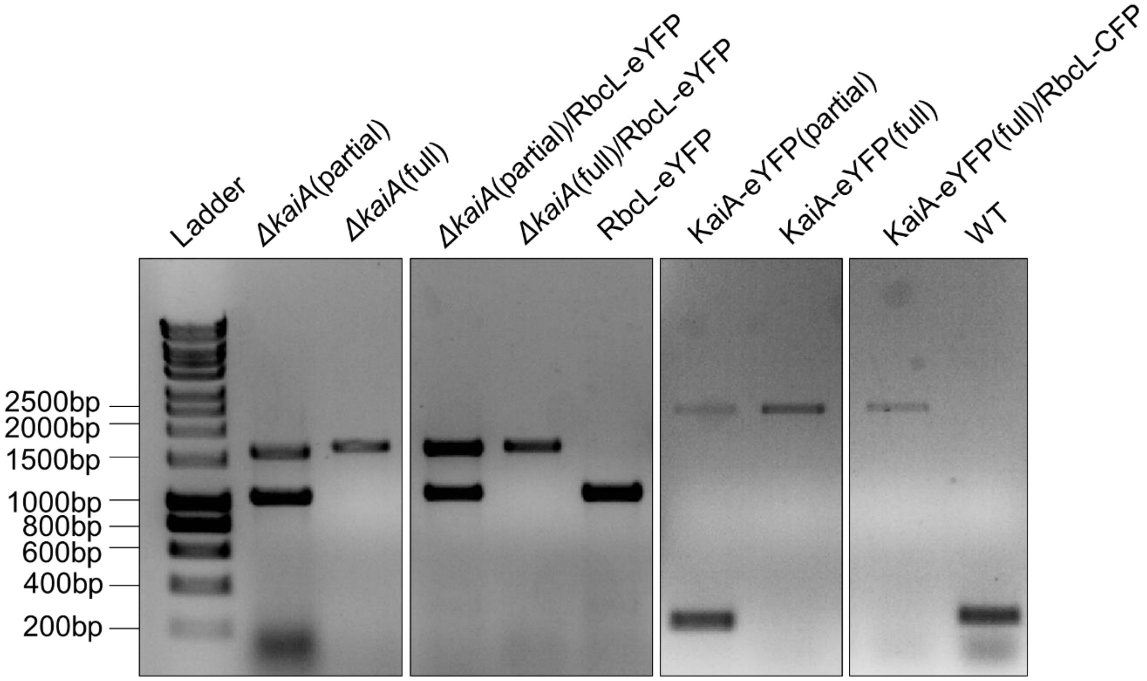

Figure S4. PCR screening of the segregation of *kaiA* mutants. The evaluation of segregation was performed by PCR using primers designed across the insertion/deletion sites (Table S2), where different sizes of band indicate non-insert or successful insertion/deletion respectively. For *ΔkaiA*, the WT band size is 1045 bp and knockout band size is 1602 bp; For KaiA-eYFP, the WT band size is 282 bp and YFP full-segregation band size is 2408 bp. Only fully-segregated strains (shown as full, partial segregation shown as partial) were studied in this work.

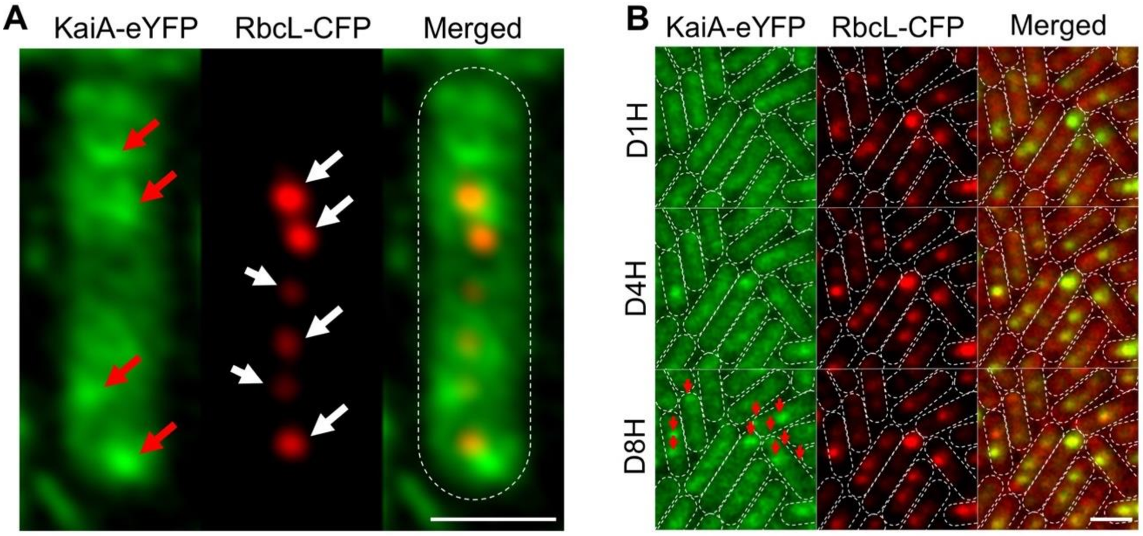

Figure S5. Fluorescence images of the KaiA-eYFP/RbcL-CFP mutant show the distribution of carboxysomes and KaiA assemblies in Syn7942. A. Representitive confocal images were taken at D8H shown both carboxysomes (white arrows) and KaiA assemblies (red arrows). Scale bar = 1 μm. B. Time-lapse images revealing the formation process of KaiA assemblies during the dark period (red arrows). Scale bar = 2 μm.

Supplementary References

1. Sun, Y.; Wollman, A.J.M.; Huang, F.; Leake, M.C.; Liu, L.N. Single-organelle quantification reveals the stoichiometric and structural variability of carboxysomes dependent on the environment. *Plant Cell* **2019**, *31*, 1648–1664.
2. Huang, F.; Vasieva, O.; Sun, Y.; Faulkner, M.; Dykes, G.F.; Zhao, Z.; Liu, L.N. Roles of RbcX in Carboxysome Biosynthesis in the Cyanobacterium *Synechococcus elongatus* PCC7942. *Plant Physiol* **2019**, *179*, 184–194, doi:10.1104/pp.18.01217.
3. Mackey, S.R.; Ditty, J.L.; Clerico, E.M.; Golden, S.S. Detection of rhythmic bioluminescence from luciferase reporters in cyanobacteria. *Methods Mol Biol* **2007**, *362*, 115–129, doi:10.1007/978-1-59745-257-1_8.
